# The Interplay of Family Dynamics, Lifestyle, and ADHD: A Case–Control Study on Sociodemographic Risk Factors

**DOI:** 10.1002/brb3.70830

**Published:** 2025-09-07

**Authors:** Hülya Tercan, Pınar Bayhan

**Affiliations:** ^1^ Child Development Department, Faculty of Health Sciences Hacettepe University Ankara Turkey

**Keywords:** attention deficit hyperactivity disorder (ADHD) risk factors, case–control study, familial and environmental influences, screen time and nutrition

## Abstract

**Purpose:**

The study aims to assess familial and environmental characteristics and daily routines (nutrition, sleep, and screen time) associated with attention‐deficit/hyperactivity disorder (ADHD) in Turkish children and compare them with typically developing peers.

**Methods:**

A case–control study was conducted with 106 ADHD‐diagnosed children and 100 typically developing peers. Data were analyzed using descriptive statistics and logistic regression models to determine risk factors for ADHD. Descriptive analyses summarized data, and logistic regression estimated ADHD associations between ADHD status and familial environmental factors.

**Finding:**

Lower parental education and employment rates, alongside increased screen time and unhealthy dietary habits, were associated with ADHD risk. Rates of hyperactivity and impulsivity were higher in parents of ADHD‐diagnosed children than in the control group (*p* < 0.01). Additionally, ADHD children had significantly higher screen time, snack, and sugar consumption compared to controls (all *p* < 0.001).

**Conclusion:**

These findings highlight the multifaceted nature of ADHD risk, emphasizing the interplay between genetic predisposition, environmental influences, and modifiable lifestyle factors. Integrating family‐based interventions and targeted public health strategies may be crucial in addressing these associated factors and supporting developmental outcomes for children with ADHD.

## Graphical abstract

This study investigates how family dynamics and lifestyle behavior, including screen time, diet, and sleep patterns, are associated with the likelihood of ADHD in children. Using a case–control design and parent‐reported data, the analysis reveals that screen exposure, dietary habits, and sleep routines are significantly linked to ADHD risk.

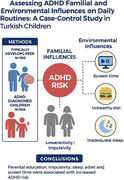

## 1 Introduction

Within the spectrum of prevalent disorders during childhood and adolescence, attention deficit hyperactivity disorder (ADHD) is frequently characterized by essential symptoms, including impulsivity, attention deficits, motor difficulties, minimal brain dysfunction, and hyperactivity, despite evolving definitions over the last five decades (Riglin et al. 2016). When examining specific data on the global prevalence of ADHD, it is estimated that the prevalence rate among children and adolescents aged 6–18 worldwide is between 5% and 7% (Polanczyk et al. 2007). A more recent meta‐analysis has updated this rate to 7.2%, indicating that approximately one in 14 children is affected by this disorder (Thomas et al. 2015).

According to the diagnostic criteria outlined in the Diagnostic and Statistical Manual of Mental Disorders‐5 (DSM‐5), attention deficits, impulsivity, and hyperactivity can impede one's ability to function across multiple environments such as home, school, or work. Symptoms typically occur in early childhood, impacting an estimated 3%–10% of school‐age children worldwide (Holden et al. 2013). According to research conducted in Turkey, the prevalence of ADHD among Turkish primary school children was found to be 8.4% in a study conducted during 2017–2018 (Ercan et al. 2013). These findings indicate that ADHD is a prevalent and significant public health issue among children in Turkey.

The study initiates a comprehensive review of the interplay between attention deficit, genetic factors, and the environment. It then synthesizes the existing literature on environmental factors most closely associated with attention deficit, primarily focusing on dietary habits (Del‐Ponte et al. 2019; Ríos‐Hernández et al. 2017), sleep (Hvolby 2015; Owens 2008), and screen time (LeBlanc et al. 2015; Lissak 2018). Finally, we present the conclusions of our study findings and summarize the methodological limitations of our research and our recommendations for possible future studies.

### 1.1 ADHD and Gene‐Environment

According to a longstanding perspective, children born to parents with a familial history of psychiatric disorders or attention‐related issues exhibit a heightened susceptibility to attention‐related problems (Franke et al. 2012; Larsson et al. 2013). Recent studies from the near past to the present underscore that children genetically predisposed to attention disorders may typically begin manifesting attention issues in adverse environmental and familial contexts (Finer et al. 2020; Nigg 2013). Despite the identification of various environmental and genetic characteristics that potentially contribute to the onset of attention deficit in children, the underlying mechanisms of these relationships remain intricate. Although certain environmental risk factors postulated in the literature may elucidate the origins of attention‐related issues, they could also manifest as outcomes of the disorder and its symptoms. The following enumerates some of the environmental factors most prominently linked to ADHD in existing literature.

### 1.2 ADHD and Nutrition

In recent years, nutrition, being a relatively modifiable environmental factor, has garnered attention as a potential intervention avenue for ADHD (Wang et al. 2021). Some studies suggest that children with ADHD may exhibit challenges in adhering to healthy eating habits compared to their non‐ADHD counterparts (Del‐Ponte et al. 2019; Ríos‐Hernández et al. 2017). Furthermore, specific dietary patterns, such as those characterized by the consumption of junk food, processed food, desserts, and fast food, have been positively associated with ADHD (Rose et al. 2021). In contrast, healthy dietary patterns featuring Mediterranean‐type vegetables and fruits, rich in micronutrients, are inversely linked to the risk of ADHD (Khoshbakht et al. 2021). These findings reinforce the notion that nutrition may play a role in influencing the risk of developing ADHD.

### 1.3 ADHD and Sleep

It has been documented that sleep issues associated with attention disorders are predominantly behavioral, often characterized by challenges in falling asleep, irregular sleep durations, difficulty waking up in the morning, fatigue, or excessive sleep (Owens 2008). Beyond these findings derived from attention‐focused sleep research, it is crucial to acknowledge that the relationship between sleep and ADHD is likely intricate and multifaceted (Becker and Gregory 2020). Sleep problems can serve as a distinctive characteristic of ADHD and may be exacerbated by specific symptoms of the disorder. Furthermore, some sleep issues may independently exhibit features resembling ADHD symptoms (Hvolby 2015; Owens 2008). In this study, to gain a deeper understanding of potential sleep‐related issues in children diagnosed with attention deficit, we queried mothers and fathers about their children's sleep routines.

### 1.4 ADHD and Screen Culture

Over the last decade, there has been a steady increase in screen time attributed to pervasive use of platforms such as the Internet, social networks, and video games (Smith and Langberg 2018; Wu et al. 2014). The advent of this “screen culture” has instigated a paradigm shift in the children's environment, prompting numerous studies in recent years to investigate its effects (Domingues‐Montanari 2017; LeBlanc et al. 2015; Lissak 2018). This raises the question of the potential effects of children's screen time on ADHD, a condition associated with an elevated risk of various psychiatric disorders (Domoff et al. 2019).

In summary, ADHD is recognized as a complex condition influenced by both genetic and environmental factors (Huang et al. 2018). In addition to its biological underpinnings, ADHD is associated with social factors such as parental education levels, family structure, and general characteristics of the children. Accordingly, this study aims to examine the familial, environmental, and daily life factors influencing ADHD by utilizing a comprehensive questionnaire that encompasses these dimensions.

## 2 Materials and Methods

This study follows the STROBE (Strengthening the Reporting of Observational Studies in Epidemiology) guidelines to ensure the transparent and comprehensive reporting of observational research findings. In the period between January and March 2023, parents of children meeting the DSM‐5 criteria for ADHD were included in our study using a stratified sampling system from seven primary schools in the capital city of Ankara, Turkey. The selection of seven primary schools in the capital city of Ankara was guided by a stratified sampling strategy to ensure representation from diverse socioeconomic backgrounds. Schools were chosen on the basis of their geographic distribution and the socioeconomic diversity of their student population to mitigate potential biases stemming from regional disparities. An a priori power analysis was conducted using GPower (version 3.1) to estimate the required sample size for detecting medium effect sizes in logistic regression analyses. Assuming an odds ratio of 2.0, a two‐tailed *α* level of 0.05, and 80% statistical power (1 − *β* = 0.80), the required total sample size was estimated at 190 participants (95 per group). Our final sample of 206 children (106 with ADHD and 100 controls) exceeds this threshold, indicating that the study was adequately powered to detect medium‐sized effects. The final sample of 106 children diagnosed with ADHD and 100 typically developing peers was deemed appropriate for robust comparisons while maintaining feasibility within the study's logistical constraints. As a control group, parents of 100 typically developing children from the same schools were randomly selected, and the parents were matched with children diagnosed with ADHD based on gender and age. Children exhibiting ADHD characteristics were previously diagnosed and reported by a child psychiatrist.

The inclusion criteria for this study were as follows: children aged 6–9 years diagnosed with ADHD by a child psychiatrist according to DSM‐5 criteria, children without intellectual or physical disabilities (IQ score ≥70), and children without severe medical conditions (e.g., diabetes and Crohn's disease), neurological disorders (e.g., epilepsy), or severe psychiatric disorders (e.g., pervasive developmental disorders). Attention was paid to ensuring that children diagnosed with ADHD did not have any other physical illnesses, psychotic disorders, or severe psychiatric disorders. Children with secondary diagnoses were excluded from the study.

### 2.1 Participants

The study was conducted with parents of children/adolescents who received a diagnosis of ADHD based on a health board report by a child/adolescent psychiatrist in Turkey, meeting the DSM‐5 criteria. The participants were between the ages of 6 and 9, without intellectual or physical disabilities (i.e., IQ score ≥70), or additional severe medical conditions (e.g., diabetes and Crohn's disease), neurological disorders (e.g., epilepsy), or severe psychiatric disorders (e.g., pervasive developmental disorders). The control group consisted of parents of typically developing children. General demographic characteristics of the participants are shown in Table 1.

**TABLE 1:** Family characteristics between the attention deficit hyperactivity disorder (ADHD) (*n *= 106) and the control (*n* = 100) groups.

Characteristics	ADHD *N* %	Control *N* %	*t*/*χ* ^2^	*p* value
	Parents (*n* = 106)	Parents (*n* = 100)		
	(mean ± SD) 32.1% ± 1.39**%**	(mean ± SD) 33.2% ± 1.59**%**	0.10	0.81
Age				
20–24 25–29 30–34 35–39 40 and over	8.5	8.0		
	31.1	25.0		
	32.1	36.0		
	19.8	24.0		
	8.5	7.0		
Sex			0.68	0.350
Female Male	62.2	65		
	37.7	35		
Education			39.91	<0.001
Primary edu. High school Undergraduate Postgraduate degree	30.2	10.0		
	36.8	40.0		
	31.1	45.0		
	1.9	5.0		
Working status			48.70	<0.001
Working				
Mother Father	42.4	58		
	83	90		
Not working			35.40	<0.001
Mother Father	57.6	42		
	16	10		
Family type			3.70a	0.14
Core family Big family Other (single parent)	77.3	76		
	16.9	18		
	5.6	6		
Family history				
Psychiatric problems Speech problems Hyperactivity/Impulsivity N/A	7.5	6		
	20.7	8	19.19^a^	<0.001
	33.9	10	6.29^a^	0.006
	37.7	76		

^a^ Fisher's exact test.

### 2.2 Data Collection Tool

The attention deficit‐related characteristics form, developed by the researcher, was utilized for investigating familial and environmental factors. This form, comprising a total of 45 items covering family environment, parental characteristics, child characteristics, screen usage, dietary habits, and sleep patterns, was provided to parents for completion. Sleep and screen time data were collected through parental reports due to practical and logistical considerations. Parents were asked to report their child's average screen time and sleep duration in hours per day based on their typical routines during the past month. Objective measurements, such as actigraphy for sleep or digital monitoring for screen time, were not feasible within the scope of this study. Parental reports, despite their inherent limitations, provide valuable insights into children's daily routines, particularly in home environments where direct observational methods may be intrusive. Responded to using multiple‐choice options by parents, this questionnaire demonstrates good reliability and validity characteristics. The Cronbach's *α* coefficient for this survey in the current study was 0.83. The inter‐subscale correlations of the utilized questionnaire are presented in Table 4.

### 2.3 Statistical Analysis

Statistical analyses were conducted using the IBM SPSS 23.0 software package. Categorical data were presented as frequency (percentage), whereas continuous data were presented as mean ± standard deviation. The normality of continuous variables was verified using Kolmogorov–Smirnov and Shapiro–Wilk statistics. Independent samples *t*‐test and one‐way ANOVA were utilized to compare scores for variables exhibiting normal distribution. Pearson correlation analysis was employed to evaluate inter‐subscale correlations. Initially, univariate logistic regression analyses were conducted to identify individual associations between ADHD and relevant predictors. Variables that showed significance in the univariate models were subsequently included in multivariable logistic regression models to control for potential confounders, including parental education level, employment status, and family history of ADHD‐related traits (e.g., psychiatric problems and hyperactivity). Variables identified as significant in univariate models were then included in multivariable logistic regression models to control for confounding. *p* values for trend were calculated from the Cochran–Mantel–Haenszel chi‐squared test. Two‐sided *p* < 0.05 was considered statistically significant. Missing data were handled using listwise deletion. The proportion of missing data was below 5% across all primary variables, and thus, no imputation methods were employed.

## 3 Results

### 3.1 General Demographic Characteristics

Parents of 106 ADHD‐diagnosed and 100 typically developing children participated in the study. ADHD parents were younger on average (32.1 vs. 33.3 years) and had significantly lower education and employment rates (*p* < 0.001; Table 1). Family structure did not differ between groups (*p* = 0.14). ADHD symptoms, hyperactivity, and speech problems were more prevalent in the childhood histories of parents in the ADHD group (*p* < 0.01).

Table 2 shows the general characteristics of children in the ADHD and control groups. Gender distribution and age were similar between the ADHD and control groups (*p *> 0.05; Table 2). Similarly, there was no significant difference between the two groups in terms of birth weeks and birth orders. Children with ADHD exhibited higher conduct problems, conflict, and hyperactivity than controls (*p* < 0.001; Table 2).

**TABLE 2:** General characteristics between the children with attention deficit hyperactivity disorder (ADHD) (*n* = 106) and the control (*n* = 100) groups.

	ADHD *N* %	Control *N* %	*t*/*χ* ^2^	*p* value
General characteristics	Children (*n* = 106)	Children (*n* = 100)		
Age	(mean ± SD) 8.28 ± 1.6	(mean ± SD) 8.0 ± 1.56	0.22	0.950
Gender			0.65	0.180
Girl Boy	%33	%35		
	%67	%65		
Birth week			0.11	0.920
≤28 weeks 29–35 weeks 36–39 weeks ≥40 weeks	0.9	3.0		
	6.6	7.0		
	63.2	66.0		
	29.2	24.0		
Birth order			45.40	0.810
Firstborn Second Third Lastborn	51.9	53		
	30.2	34		
	15.1	11		
	2.8	2.0		
Conduct problem	87.5	39	86.38	<0.001
Conflict	73.1	43	78.10	<0.001
Hyperactivity	77.7	20	70.63	<0.001

When Table 3, which presents the general routines of children diagnosed with ADHD and the control group regarding nutrition, screen time, and sleep, is examined, no significant difference was found between the two groups in terms of duration of breastfeeding during infancy (*p* > 0.05; Table 3). However, there is a significant difference between children diagnosed with ADHD and the control group in terms of junk food and sugar consumption (*p* < 0.001; Table 3). Both junk food consumption and sugary food consumption rates are higher in the ADHD group compared to the control group (both *p* < 0.001; Table 3). In terms of screen time, both TV viewing time and tablet or smartphone usage rates are significantly higher in children diagnosed with ADHD compared to the control group (both *p* < 0.001; Table 3). Sleep and wake‐up times of children, as reported by parents, are shown in Table 3. Although bedtime did not differ, ADHD children had significantly more difficulty waking up in the morning (*p* < 0.001; Table 3).

**TABLE 3:** Basic habits and consequences of children with attention deficit hyperactivity disorder (ADHD) and the control group.

	ADHD *N* %	Control *N* %	*t/χ* ^2^	*p* value
Nutrition habits	Children (*n* = 106)	Children (*n* = 100)		
Breast milk time	%	%	2.70	0.16
None <6 months 6–12 months 13–18 months 18–24 months	8.5	6.6		
	21.7	13.2		
	17.9	11.3		
	30.2	20.8		
	21.7	15.1		
Junk food consump.			40.91	<0.001
None Daily Once in 2 days Once a week	0.9	0.0		
	50.0	31.1		
	39.6	28.3		
	9.4	7.5		
Sugary food consump.			34.69	<0.001
None Daily Once in 2 days Once a week	1.9	0.9		
	55.7	35.8		
	37.7	27.4		
	4.7	2.8		
Technological habits (hours/day)				
TV time			40.81	<0.001
≤1 h 2–3 h 4–5 h 6–7 h >8 h	14.2	5.7		
	39.6	28.3		
	23.6	17.0		
	13.2	9.4		
	9.4	6.6		
Tablet/Smartphone use			11.60	0.001
Uses Not using	79.2	51.9		
	20.8	15.1		
Tablet/Smartphone time			28.80	<0.001
≤1 h 2–3 h 4–5 h 6–7 h >8 h	24.5	15.1		
	34.0	23.6		
	30.2	19.8		
	4.7	2.8		
	6.6	5.7		
Sleep habits (hours/day)				
Night sleep time ≤5 h 6–8 h 9–11 h >12 h			5.34	0.060
	0.9	3		
	24.5	40		
	69.8	40		
	4.7	17		
Difficulty waking up in the morning				
	79.5	12.3	132.11	<0.001

Table 4 presents the interdimensional correlations among the subscales of the ADHD‐related characteristics questionnaire, as well as their associations with the total score. Significant positive correlations were observed between the family environment, parental characteristics, child characteristics, screen time, and nutrition/sleep dimensions, indicating the interconnected nature of these factors in the context of ADHD. Notably, screen time demonstrated a strong correlation with both child characteristics (*r* = 0.334, *p* < 0.01) and parental characteristics (*r* = 0.493, *p* < 0.01), suggesting a potential bidirectional influence between parental behaviors and children's screen exposure. Furthermore, the highest correlation was found between total questionnaire scores and screen time (*r* = 0.900, *p* < 0.01), highlighting the significant role of digital engagement in ADHD‐related behavioral patterns. These findings reinforce the importance of considering multiple environmental and familial factors in ADHD research and intervention strategies.

**TABLE 4:** The interdimensional correlations and the correlations between the dimension‐total score of attention deficit hyperactivity disorder (ADHD)‐related characteristics questionnaire (*N* = 106).

Subdimensions	Family environment	Parental characteristics	Child characteristics	Screen time	Nutrition and sleep
**Family environment**	—	0.525^**^	0.086	0.372^**^	0.110^**^
**Parental characteristics**		—	0.090	0.493^**^	0.211^**^
**Child characteristics**			—	0.334^**^	0.229^**^
**Screen time**				—	0.335^**^
**Nutrition and sleep**					—
**Total**	0.640^**^	0.622^**^	0.559^**^	0.900^**^	0.718^**^

** *p* < 0.01.

**FIGURE 1:**
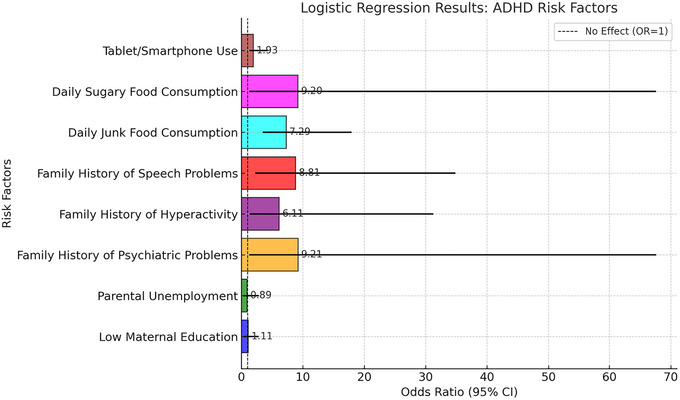
Odds ratios for risk factors associated with ADHD (95% CI), derived from multivariable logistic regression models adjusted for maternal education level, parental employment status, and family psychiatric history. ADHD, attention deficit hyperactivity disorder.

In addition to these results, unconditional logistic regression analysis was conducted to examine the ADHD risk associated with familial and environmental factors. The final multivariable logistic regression models presented in Table 5 controlled for key sociodemographic and familial confounders, including maternal education level, parental employment status, and history of psychiatric or hyperactivity symptoms in family members. Table 5 presents the results of multivariable logistic regression models in which each factor was adjusted for key confounders, including maternal education level, parental employment status, and family psychiatric history. These adjusted models provide estimates of the independent association between each variable and ADHD risk. As shown in Table 5, a lower maternal education level and parental unemployment were associated with an increased likelihood of ADHD. Specifically, children whose mothers had only a high school or primary school education had significantly higher odds of ADHD (OR = 8.78, 95% CI: 2.12–31.73; OR = 9.11, 95% CI: 1.20–65.62, respectively). Parental unemployment was not significantly associated with ADHD risk (OR = 0.89, 95% CI: 0.30–2.82). Additionally, psychiatric problems, hyperactivity/impulsivity, and speech problems in family history emerged as significant risk factors for ADHD, with OR values of 9.21 (95% CI: 1.25–67.62), 8.81 (95% CI: 2.23–34.83), and 6.11 (95% CI: 1.34–31.23), respectively. Dietary habits also played a crucial role: Frequent consumption of junk food was significantly associated with higher ADHD risk (e.g., daily junk food: OR = 7.29, 95% CI: 3.44–17.93), as was sugary food intake (e.g., daily consumption: OR = 9.20, 95% CI: 1.25–67.61). Furthermore, as illustrated in Figure 1, excessive screen exposure, particularly the use of tablets and smartphones, was identified as a contributing factor to ADHD risk (OR = 1.93, 95% CI: 1.28–4.27). Together, these findings emphasize the multifaceted nature of ADHD risk, highlighting the interplay between genetic predisposition, lifestyle factors, and environmental influences.

**TABLE 5:** Multivariable logistic regression analyses of factors associated with attention deficit hyperactivity disorder (ADHD) risk (adjusted for parental education, employment status, and family psychiatric history).

	*B*	Wald statistic	*p* value	OR (95% CI)	*p* for trend
Mother education				1.00 (Ref)	<0.001
Postgraduate degree Undergraduate High school Primary edu.					
	0.15	0.14	0.626	1.11 (0.39–2.81)	
	1.57	9.5	0.001	2.22 (0.93–5.22)	
	2.14	4.7	0.021	8.78 (2.12–31.73)	
	2.23	3.2	0.011	9.11 (1.20–65.62)	
Working status					<0.001
Not working	−0.104	0.030	0.847	0.89 (0.30–2.82)	
Family history				1.00 (Ref)	<0.001
Psychiatric problems Speech problems Hyperactivity/Impulsivity	2.19	5.454	0.064	9.21 (1.25–67.62)	
	1.24	6.459	0.002	6.11 (1.34–31.23)	
	2.24	13.141	<0.001	8.81 (2.23–34.83)	
				
Junk food consump.				1.00 (Ref)	<0.001
None Daily Once in 2 days Once a week					
	−0.186	0.132	0.501	0.73 (0.18–2.22)	
	2.22	19.8	0.003	7.29 (3.44–17.93)	
	1.11	2.8	0.002	2.49 (0.88–5.33)	
	1.12	5.7	0.029	3.66 (1.62–7.85)	
Tablet/Smartphone use					0.001
Uses vs. not using	0.645	4.109	0.028	1.93 (1.28–4.27)	
Sugary food consump.				1.00 (Ref)	<0.001
None Daily Once in 2 days Once a week	0.147	4.674	0.003	1.12 (0.49–2.82)	
	2.12	0.122	0.726	9.20 (1.25–67.61)	
	2.18	9.639	0.027	8.80 (2.22–35.83)	
	0.186	4.546	0.017	2.66 (1.34–57.85)	
Difficulty waking up in the morning					<0.001
	79.5	12.3	132.11	<0.001	

## 4 Discussion

In this study, aiming to address factors related to both familial and environmental perspectives as well as daily routines associated with attention deficit, data from 106 children diagnosed with ADHD and 100 typically developing children were collected from their parents and analyzed.

### 4.1 Demographic Characteristics

Our findings indicate that parents of children diagnosed with ADHD had lower education levels and employment rates than those in the control group. Logistic regression analysis revealed a trend linking lower parental education and unemployment to an increased risk of ADHD, suggesting that families of children with ADHD may experience greater financial difficulties and lower socioeconomic status (Chou et al. 2020). In addition to its direct impact on ADHD prevalence, socioeconomic adversity may act as a confounding variable that influences family routines and lifestyle behaviors. Children from lower SES backgrounds may be more exposed to environmental stressors, limited access to nutritious food, and unstructured screen time, which are themselves associated with neurodevelopmental outcomes. Nationally representative studies show that socioeconomic disadvantage is consistently linked to both elevated ADHD risk and poorer health‐related behaviors in children (Evenson and Simon 2005; Russell et al. 2016).

The lower maternal employment rate in the ADHD group may reflect challenges in balancing workforce participation with their children's educational and behavioral needs. Socioeconomic stressors can exacerbate parental anxiety and contribute to increased behavioral difficulties in children (Machlin et al. 2020; Rowland et al. 2018; Wallis et al. 2008).

Additionally, ADHD symptoms were more prevalent among parents in the ADHD group, with higher reported rates of hyperactivity and speech problems in their childhood histories. This aligns with prior research highlighting the genetic predisposition to ADHD, as parental ADHD symptoms may contribute to symptom transmission across generations (Hawi and Samaha 2019). Furthermore, parent‐reported assessments indicated that children with ADHD exhibited significantly higher rates of conduct problems, conflict, and hyperactive behaviors than their peers, reinforcing the presence of behavioral challenges both at home and in school settings.

### 4.2 Nutrition Habits

Our study has provided striking data regarding the dietary habits of children with ADHD. Our results indicate that children experiencing attention deficit exhibit a significantly high frequency of consuming snacks and sweets almost every day. There is a significant difference between children diagnosed with ADHD and the control group in terms of snack and sweet consumption. In the ADHD group, both snack and sweet consumption rates are higher compared to the control group. Additionally, the unconditional regression model revealed that an increase in snack and sweet consumption habits may escalate the risk of ADHD.

Supporting our findings, the research on the relationship between attention and eating patterns in children emphasized that the consumption of snacks and junk foods is higher in children with attention problems and that these children are likely to demonstrate excessive eating behavior (Hartmann et al. 2010; Yan et al. 2018). In addition, the literature hosts findings that adults with apparent attention deficit characteristics are at more risk of obesity since they more frequently consume snacks and added sugar compared to typical people (Breton et al. 2021; Cortese et al. 2016; Benton 2008; Wiles et al. 2009). Besides, this causality may be explained by the idea that children with both attention deficit and hyperactivity may need more energy than their less active peers and, therefore, have high glucose intakes to satisfy their energy needs.

### 4.3 Sleep‐Related Characteristics

Our findings regarding the sleep routines of children with ADHD indicated that although there was no significant difference between the ADHD group and the control group in terms of nighttime sleep onset, children in the ADHD group experienced more difficulty waking up in the morning compared to the control group.

A meta‐analysis by Cortese et al. (2009), covering subjective and objective research on sleep and ADHD, revealed significant associations between various sleep disorders and ADHD, except for sleep duration (Cortese et al. 2009). The authors speculated that this result might be attributed to the participating parents’ different perspectives of sleep duration (e.g., assuming a child is asleep from the time the lights are turned off to the waking‐up time), which may also apply to our results on sleep and other environmental factors. Overall, it is evident that people with attention problems experience more variability in their sleep patterns, which may need further investigation.

### 4.4 Screen Time

The literature is almost full of cross‐sectional and longitudinal research on ADHD that explored the relationships between screen time and symptoms of hyperactivity, attention deficit, or externalizing behavior (Ra et al. 2018; Tamana et al. 2019; Xie et al. 2020). Although the findings of most of these studies (Lingineni et al. 2012; Tamana et al. 2019; Xie et al. 2020) showed that screen time as an environmental factor is associated with increased externalizing behavior, hyperactivity, or attention deficit symptoms in healthy preschool and school‐age children, the literature also hosts controversial results. However, screen time appeared as a robust environmental factor that may be associated with attention deficit in our study.

In our study, we found that both TV viewing time and the use of tablets or smartphones were significantly higher in children diagnosed with ADHD compared to the control group (*p* < 0.001). Our regression analysis indicated that increased screen time is associated with a higher risk of ADHD (OR [95% CI] = 1.93 [1.28–4.27]). This finding is consistent with existing literature, which suggests that excessive screen time can exacerbate behavioral problems such as hyperactivity, inattention, and impulsivity (LeBlanc et al. 2015; Lissak 2018). It is important to note that, due to the retrospective nature of the case–control design, the observed associations cannot be interpreted as evidence of causality. The reported odds ratios reflect statistical associations between exposures and ADHD status.

The association between excessive screen time and ADHD symptoms may be explained by several interrelated mechanisms. From a behavioral perspective, prolonged screen engagement can reduce opportunities for self‐directed play, interpersonal interaction, and sustained attention, all of which are essential for the development of executive functions (Swing et al. 2010). Neurobiologically, repeated exposure to highly stimulating digital content may increase reward sensitivity and impulsive decision‐making, as these platforms often activate dopaminergic reward circuits associated with immediate gratification (Montag and Walla 2016). Furthermore, increased screen exposure, particularly during evening hours, can disrupt circadian rhythms and melatonin secretion, leading to sleep disturbances that may further exacerbate attention and behavior regulation difficulties (Paulus et al. 2018). These interacting factors suggest that screen time may not merely co‐occur with ADHD symptoms but actively contribute to the cognitive‐emotional load experienced by vulnerable children.

Although previous research presented many possible explanations for the length of children's screen time, we may emphasize that parental perceptions may be influential on our findings of screen time. Many of our participating parents may have perceived and reflected the time their children spend in front of the screen as more than it actually is. Despite an apparent controversy between previous research findings and parent‐report information, most recent studies still mention the detrimental effects of electronic devices on children.

Another potential source of confounding is parental psychopathology, including ADHD‐related traits such as impulsivity, inattention, or emotional dysregulation. Such traits may influence parenting practices, household structure, and child routines, while also introducing bias in parent‐reported data. Prior research has demonstrated that maternal ADHD symptoms are associated with inconsistent parenting and increased behavior problems in children with ADHD (Chronis‐Tuscano et al. 2008). Moreover, parental stress and emotional dysregulation can further impair parental monitoring of screen time and routines (Bernstein et al. 2010).

## 5 Conclusion

Many clinical studies have been conducted on the gene‐environment interactions of ADHD over the years. The current research is a case–control study based on epidemiological investigation among primary school children in Turkey. The findings of this study suggest that considering factors such as general demographic characteristics of parents and children, as well as lifestyle changes, including nutrition, screen time, and sleep, may be important indicators for improving the functional outcomes of children.

Given the strong associations identified between ADHD and modifiable environmental factors such as nutrition, screen time, and sleep patterns, targeted interventions should be developed to mitigate these risks. Clinically, healthcare professionals should incorporate lifestyle assessments into ADHD screening and management, emphasizing parental education on healthy dietary habits, screen time regulation, and sleep hygiene practices. Additionally, integrating psychoeducation programs for parents into ADHD treatment plans may enhance family awareness and improve child outcomes. From a policy perspective, public health initiatives should prioritize structured guidelines on screen time for children, nutritional education in schools, and broader accessibility to early intervention services. Regulatory frameworks encouraging responsible media consumption and school‐based interventions addressing ADHD risk factors may be crucial in addressing these associated factors and supporting developmental outcomes of ADHD in childhood populations. Future research should explore the effectiveness of such policies in diverse cultural and socioeconomic contexts.

### 5.1 Limitations and Future Directions

This case–control study recruited participants from public elementary schools, ensuring that the ADHD sample reflects general population characteristics. Control group participants were selected from the same schools and classes, enhancing the validity of comparisons. Although our findings align with previous research on ADHD's impact on children and their families, direct comparisons remain challenging due to variations in study designs. Additionally, potential interaction effects between familial and lifestyle factors (e.g., parental ADHD history × screen time) were not tested in the current regression models due to sample size constraints and the risk of overfitting. Future studies with larger and more powered samples should investigate such interactions to uncover potential moderating effects and refine risk models for ADHD.

Despite its strengths, this study has several limitations. First, the lack of blinding may introduce potential bias, as the researcher was partially aware of diagnostic status. Second, reliance on parent‐reported measures for screen time, sleep, and nutrition introduces recall and social desirability biases. However, these measures provide ecologically valid insights into children's daily routines in naturalistic home settings, especially where observational or digital tools are not feasible. Future studies should incorporate objective assessments, such as actigraphy for sleep and digital monitoring for screen use, to validate and extend our findings. Third, the data collection relied exclusively on parent‐report measures, which introduces potential reporting bias, particularly for behaviors like screen time, diet, and sleep. Parental perceptions may not always accurately reflect children's actual routines and could be influenced by recall or social desirability biases. Fourth, objective assessment tools such as actigraphy for sleep or digital logs for screen use were not employed due to logistical and financial constraints, which limits the precision of behavioral measurements. Fifth, although all ADHD diagnoses were clinically verified, information on the children's medication use was not systematically collected or controlled for in the analyses. Given that psychostimulant medications can influence sleep, appetite, and behavior, this remains a relevant limitation that future studies should address. Furthermore, data on medication use among children with ADHD were not available. As stimulant medication can influence factors such as sleep, appetite, and behavioral regulation, the lack of adjustment for medication status represents an important limitation.

Nonetheless, our findings provide valuable insights into the role of environmental factors, family history, and lifestyle in ADHD. Although much of the existing research focuses on pharmacological treatments, our results highlight the potential benefits of family‐centered interventions in guiding children toward healthier routines. Future research should prioritize longitudinal and experimental designs to better establish causality and refine intervention strategies.

## Author Contributions

**Hülya Tercan**: conceptualization, writing – original draft, methodology, validation, visualization, data curation. **Pınar Bayhan**: writing – review and editing, project administration, supervision.

## Conflicts of Interest

The authors declare no conflicts of interest.

## Peer Review

The peer review history for this article is available at https://publons.com/publon/10.1002/brb3.70830

## Data Availability

The data that support the findings of this study are available from the corresponding author upon reasonable request.
